# A method for deconvolution of integrated electronic portal images to obtain incident fluence for dose reconstruction

**DOI:** 10.1120/jacmp.v6i4.2104

**Published:** 2005-11-22

**Authors:** Wendel Dean Renner, Kevin Norton, Timothy Holmes

**Affiliations:** ^1^ Math Resolutions LLC 5975 Gales Lane Columbia Maryland 21045‐3841; ^2^ Hartford Hospital Dept. Radiation Oncology 80 Seymour Street Hartford Connecticut 06102; ^3^ The University of Connecticut Health Center Dept. Radiation Oncology Room CG178, 263 Farmington Avenue Farmington Connecticut 06030‐2930; ^4^ St. Agnes Health Care Dept. Radiation Oncology 900 Caton Avenue Baltimore Maryland 21229 U.S.A.

**Keywords:** radiation therapy quality control, IMRT, EPID

## Abstract

A method to convert integrated electronic portal imaging device (EPID) images to fluence for the purpose of reconstructing the dose to a phantom is investigated here for simple open fields. Ultimately, the goal is to develop a method to reconstruct the dose to patients. The EPID images are transformed into incident intensity fluence by spatial filtering with a deconvolution kernel. The kernel uses a general mathematical form derived from a Monte Carlo calculation of the point spread function of an EPID. The deconvolution kernel is fitted using a downhill search algorithm that minimizes the difference between the reconstructed dose and the dose measured in water. The beam profile “horns” that are removed by the EPID calibration procedure are restored to the resulting images by direct multiplication using the measured in‐air off‐axis ratio. Applying the fitted kernel to an EPID image provides the incident fluence for that beam. This beam fluence is then entered into an independent dose calculation algorithm for phantom or patient dose reconstruction. The phantom dose was computed to an accuracy of 2.0% of the $d_{\max}$dose at one standard deviation. The method is general and can possibly be applied to any EPID equipped with an integration mode. We demonstrate the application of the fitted kernel in two clinical IMRT cases.

PACS numbers: 87.52.Df, 87.53.Bn, 87.53.Dq, 87.56.Fc, 87.66.Pm

## I. INTRODUCTION

**Figure 1:**
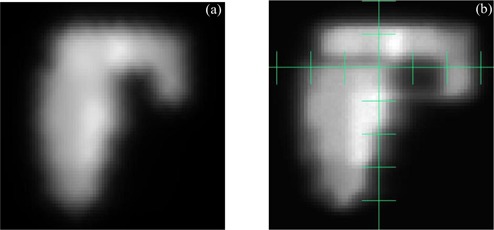
(a). Planning system QA phantom dose intensity map at $d_{\max}$for an IMRT beam. (b) Fluence image derived from X‐ray film for the *delivered* beam shown in Fig. 1(a). The scale is 1 cm per division.

**Figure 2:**
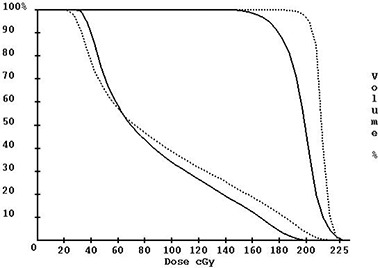
Dose‐volume histogram of the planning tumor volume (curves on the right) and the adjacent left eye (curves on the left). The dotted lines are from the planning system, and the solid lines are from the 3D dose reconstruction method.^(1)^

Intensity‐modulated radiation therapy (IMRT) has created demands for new methods of treatment verification. An example of the necessity for IMRT quality assurance (QA) is demonstrated in Fig. 1(a), which shows the QA phantom dose for an IMRT beam. Figure 1(b) shows the fluence image from the same beam as it is delivered by the LINAC. This fluence image was derived from an X‐ray film using a process described in a prior report.^(1)^ Just above the horizontal axis there is an obvious region of reduced dose as compared to that planned. Furthermore, this discrepancy occurred in two of the four beams for this plan. A dose‐volume histogram comparison (Fig. 2) of the planned and delivered beams demonstrates how the delivery system has degraded the target volume dose. Without 3D dose reconstruction, the cumulative effects of these individual beam differences cannot always be appreciated.^(^ ^2^ ^,^ ^3^ ^)^ Directly comparing the *delivered* dose to the *planned* dose on the patient's CT dataset completes the dose verification feedback loop.^(^ ^1^ ^–^ ^3^ ^)^ Dose reconstruction using the *patient's* clinical treatment beams improves the accuracy of this process. With 3D dose reconstruction, the dose delivered to the patient is demonstrated; hence, verification does not depend upon the diligence of the medical physicist to find possible errors while checking each step of the treatment‐planning and delivery process.^(1)^

Electronic portal imaging devices (EPIDs) have been investigated for the purpose of treatment verification^(^ ^2^ ^–^ ^26^ ^)^ While several reports have considered the stability and accuracy of using an EPID for dosimetric purposes,^(^ ^23^ ^–^ ^25^ ^)^ there are generally four different ways of using the EPID for dose verification. One method is to compute the dose to the EPID for each beam to compare to the EPID image of each beam.^(^ ^4^ ^–^ ^15^ ^)^ A second method is to use the EPID to verify the leaf positions for intensity‐modulated fields.^(^ ^16^ ^–^ ^18^ ^)^ A third method considers reconstructing the dose to the patient using the exit image acquired during treatment.^(^ ^19^ ^–^ ^22^ ^)^ Finally, prior reports^(^ ^2^ ^,^ ^3^ ^)^ have considered using a fourth method^(1)^ that converts the EPID image to an incident fluence distribution and uses this fluence distribution as input to a dose algorithm, which comrithm putes the dose to the patient. This method has several advantages over using the exit dose. Use of the exit dose requires subtracting the scatter generated within the patient that reaches the imaging device. This fluence must then be traced back through the patient to derive the incident fluence, which is then used to compute the dose to the patient. In addition, the patient support system may be in the exit path of the beam. Deriving the incident fluence from EPID measurement without the patient avoids such difficulties and, therefore, may be more accurate and reliable.

Renner et al.^(1)^ demonstrated how X‐ray film could be used to measure the incident fluence for patient dose reconstruction. However, using an EPID for this purpose can eliminate X‐ray film's unfavorable properties: (1) the inconsistencies inherent to film production and processing, (2) the need to handle, process, and digitize each film, and (3) the need to re‐enter the treatment room to set up, mark the central axis, and acquire each film image. Over time, the EPID should be more stable than film.^(^ ^22^ ^–^ ^24^ ^)^ In using the EPID for this technique, the couch is rotated out of the way, and the EPID is simply irradiated with the patient's clinical treatment beams. Here, we present a method to convert EPID images to an incident fluence map. We propose a simple solution for restoring the beam profile “horns” that are removed by the EPID calibration procedure. The kernel needed for this method is fitted to a known mathematical form rather than computed from first principles. To validate this dose reconstruction method, computed dose (absolute dose and relative dose profiles) is compared to measured water phantom dose.

## II. METHODS AND MATERIALS

An Elekta Precise accelerator with an iViewGT amorphous silicon flat panel imaging system and version 3.1 software was used in this investigation (Elekta AB, Stockholm, Sweden). Integration is accomplished by summing frames during the beam‐on time. Dark current for each frame is subtracted, and the pixels are divided by a full‐field flood image. The result is multiplied by a scaling factor to produce a displayable image with pixels stored as a 16‐bit number. Within a given image, unprocessed pixel values are inversely proportional to dose. Version 3.1 of the iViewGT software provides access to the pixel scaling factor, which, when divided into the pixel values of a corresponding image, produces the integrated result for the beam‐on time. However, this integration is only available for a single beam on‐off cycle. Segmented fields used in intensity modulation require a separate integrated image and pixel scaling factor for each segment. Further, the pixel scaling factor is only available on the screen through the user interface. As a result, the user must manually record the pixel scaling factor for each image and then re‐enter that number into the system used here. For the clinical application of dose reconstruction to the patient, it may be considered too tedious to assemble all the segmented image files with their respective scaling factors for routine clinical use. However, this process was sufficient to convert the integrated images to an incident fluence distribution for simple open fields and to demonstrate the feasibility of clinical use.

The EPID image size is limited to $25.6\times 25.6\;{\text{cm}}^{2}$, with a pixel size of $0.025\times 0.025\;{\text{cm}}^{2}$. Points outside of a 25.6 cm square cannot be accurately computed because a fluence measurement is not available for those points. Pencil beams outside of the measurement area are assigned a value of zero. Given that some margin around a field is also needed to account for the penumbra, clinical use will be limited to field sizes of the order of $20\times 20\;{\text{cm}}^{2}$ and smaller.

The Dosimetry Check program (Math Resolutions, LLC, Columbia, MD, U.S. patent 6,853,702, http://MathResolutions.com) was used in this study. This program reconstructs the dose from the derived incident fluence distribution images and provides tools to compare the reconstructed dose to the planned dose.

### A. EPID integration

The first investigation was to determine whether the iViewGT system could indeed integrate. Integrated pixel values are computed by subtracting the raw image pixel values from the number 65 535 $\left(2^{16}-1\right)$ and then dividing by the pixel scaling factor for that image. Five images of a $10\times 10\;{\text{cm}}^{2}$ field were acquired using monitor units of 2, 5, 50, 100, and 200. Corresponding integrated pixel values should fall on a line that intercepts the origin. To test the stability of the EPID system, this relationship was verified six weeks after the initial measurements.

### B. Calibration of EPID images to relative monitor units

For a $10\times 10\;{\text{cm}}^{2}$ reference field, a plot of integrated central axis pixel values versus monitor units yields a straight line (results from Section A). Since the reference field size for the LINAC is $10\times 10\;{\text{cm}}^{2}$, this line fit provides a means to relate integrated pixel values to the output of the LINAC. Using this line fit, all EPID pixel values are converted to a unit termed the relative monitor unit (RMU), defined previously^(1)^ as the number of monitor units that produce the same signal at the center of the reference field. In this report, the signal is the integrated pixel value. The RMU is the means by which EPID images are calibrated and renormalized for dose reconstruction.

### C. Conversion of EPID images to incident fluence

**Figure 3:**
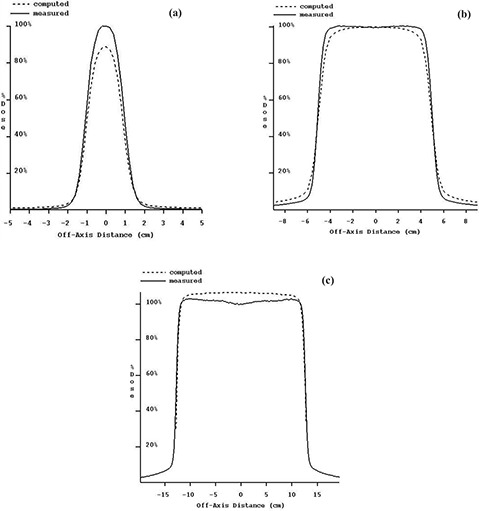
(a) to (c). Measured dose compared to computed dose (reconstructed from iViewGT integrated images *without* deconvolution and OAR correction) and renormalized to 100% of the measured dose at $d_{\max}$. Beam profiles are for 6‐MV photons at $d_{\max}=1.7\;\text{cm}$. (a) $2\times 2\;{\text{cm}}^{2}$ field size. (b) $10\times 10\;{\text{cm}}^{2}$ field size. (c) $25\times 25\;{\text{cm}}^{2}$ field size

Accurate dose computation using EPID images requires a further transformation of the RMU‐calibrated images. Figures 3(a) to (c), which show the RMU‐calibrated but otherwise uncorrected EPID‐based reconstructed dose, demonstrate the need for deconvolution and further manipulation. First, Figs. 3(a) to (c) show that the dose is wrong. The computed dose is 12.5% low for the $2\times 2\;{\text{cm}}^{2}$ field and 6.3% high for the $25\times 25\;{\text{cm}}^{2}$ field. The central axis dose for the $10\times 10\;{\text{cm}}^{2}$ field size agrees due to the method of calibration, using the $10\times 10\;{\text{cm}}^{2}$ field size as the reference. Early in our investigation we measured EPID scatter factors, $S_{\text{EPID}}$. Table 1 summarizes the various measured scatter factors for the Elekta Precise used in this study. Here we see that $S_{\text{EPID}}$, compared to $S_{c}$ and $S_{c,p}$, follows the same under‐ and overresponding trends for small and large field sizes demonstrated in Figs. 3(a) to (c). Other authors have reported both similar^(22)^ and dissimilar^(26)^ results for liquid‐filled ionization chamber EPIDs.

**Table 1:** Measured scatter factors for Elekta Precise 6‐MV photons: collimator $\left(S_{c}\right)$, water phantom $\left(S_{c,p}\right)$, and $\text{iViewGT}\;\text{EPID}(S_{\text{EPID}})$. $S_{\text{EPID}}$ was calculated using the integrated central axis pixel values for the respective field‐size images.

Fieid size $\left({\text{cm}}^{2}\right)$	$S_{c}$	$S_{c,p}$	$S_{\text{EPID}}$
2×2	0.8947	0.8859	0.8449
5×5	0.9704	0.9511	0.9316
10×10	1.0000	1.0000	1.0000
20×20	1.0307	1.0489	1.0706
25×25	1.0373	1.0598	1.0896

Second, Fig. 3(b) shows that the computed shoulder is rounded off compared to the dose profile measured in water. This indicates that the EPID system acts like a low pass filter, as one would expect with a point spread function. In the previous study using the X‐ray film technique,^(1)^ similar results were noted when solid water was used as the electron filter, as opposed to a thin sheet of copper.

And third, it is evident from the profile for the $25\times 25\;{\text{cm}}^{2}$ field that the effect of the field‐flattening filter has been removed. The EPID system is calibrated with an X‐rays off measurement and then with a full‐field flood measurement. The full‐field flood is used to normalize the response of all the pixels (similar to the Varian EPID^(3)^). This process removes the “horns” from the beam profile.

Thus, to accurately compute the dose using the Elekta iViewGT EPID, the RMU‐calibrated images must be transformed to provide the correct incident fluence. For the Varian aS500 EPID, Warkentin et al.^(2)^ computed a point spread function (kernel) that combined separate dose and glare kernels. The dose kernel accounts for dose deposition in the EPID's scintillator screen, while the glare kernel characterizes the optical photon spreading from the screen to the photodiode layer.^(2)^ (The Elekta iViewGT EPID has a similar design.) They reported that a glare kernel can be approximated by the sum of two exponentials and that the composite (dose and glare) kernel can be approximated by the convolution of the dose and glare kernels. In another report by Steciw et al.^(3)^ the point spread function was represented by a sum of exponentials. This result is used in the present study.

However, here we assume that the composite kernel of any EPID system can be represented by a sum of exponential functions, given here by

(1) $$k(r)=\sum_{i}^{n}a_{i}e^{-b_{i}r},$$

where *k(r)* is the composite point spread kernel, *r* is the radius (cm) of a point from the central axis at a source‐to‐axis distance of 100 cm, and $a_{i}$ and $b_{i}$ are parameters for each of a sum of *n* exponentials. Since *k(r)* is circularly symmetrical, its 2D frequency transform is therefore also circularly symmetrical and is given by the Hankel transform of the 1D circularly symmetrical point spread kernel as a function of radius^(27)^

(2) $$K(q)=\sum_{i}^{n}a_{i}\frac{2\pi b_{i}}{{\left(4\pi^{2}q^{2}+b_{i}^{2}\right)}^{3/2}},$$

where *q* is the frequency radius (cycles/cm) in the frequency domain. As long as $a_{i}>0$ and $b_{i}>0$, the kernel and its transform are always positive, nonzero numbers.

To convert the EPID images to incident fluence (after converting pixel values to RMUs), it is proposed to take the 2D Fourier transform (using the fast Fourier transform) of the images, divide each frequency component by the corresponding value in Eq. (2), and then transform the image back, a deconvolution process. As noted above, the value in Eq. (2) is always a positive nonzero number as long as $a_{i}>0$ and $b_{i}>0$. Restricting the fit of the kernel to positive parameters insures mathematical feasibility. Any amplification of noise at high spatial frequencies must be accepted here.

Next, each pixel in the image (at distance *r* (cm) from the central axis) is multiplied by its corresponding in‐air off‐axis ratio (OAR). The in‐air OAR was measured by scanning (Scanditronix‐Wellhofer, Bartlett, TN) the diagonals of a $40\times 40\;{\text{cm}}^{2}$ field in air with a buildup cap. Off‐axis ratios from each half of both diagonal profiles were used to calculate an average OAR. Any small error produced by the presence of the buildup cap is necessarily accepted here. Thus, OAR multiplication restores the “horns” to the EPID‐derived incident fluence.

### D. Fitting of kernel parameters

The kernel parameters, $a_{i}$ and $b_{i}$, are fitted properly when the output of our process, the *calculated* dose to a water phantom, agrees (to within 2%) with the *measured* dose to a water phantom. (Since direct measurement of intensity fluence is difficult, comparison of EPID‐derived fluence to measured fluence was avoided.) Also, since the deconvolution process is separate from the RMU calibration process, kernel parameter fitting need only be done once per photon energy. Thus, should the EPID performance change, the kernel fit is unaffected, and only the RMU calibration curve needs to be updated.

For field sizes of $2\times 2\;{\text{cm}}^{2},\;5\times 5\;{\text{cm}}^{2},\;10\times 10\;{\text{cm}}^{2},\;20\times 20\;{\text{cm}}^{2}$, and $25\times 25\;{\text{cm}}^{2}$, 6‐MV photon profiles were acquired at depths of 1.7 cm, 5 cm, 10 cm, and 20 cm at 100 cm source‐to‐surface distance (SSD) using a Wellhofer scanning system (Scanditronix‐Wellhofer, Bartlett, TN) using Wellhofer CC13 ion chambers (inner diameter 0.6 cm, volume 0.13 cm^3^). For both cross‐plane and in‐plane scans, data were acquired at 0.2‐cm increments that resulted in a total of 4280 points for the 6‐MV fit. The scan data were converted to dose (cGy) by normalizing each dataset to the measured output factor for the particular field and monitor units used for the corresponding EPID image. A downhill search algorithm was used to fit the point spread kernel parameters so as to minimize the variance between computed and measured dose, where

(3) $$\text{Variance}=\sum_{i}^{m}\frac{{\left(\text{measured}\;{\text{dose}}_{i}-\text{computed}\;{\text{dose}}_{i}\right)}^{2}}{m}$$

for all the *m* data points pooled together.

The downhill search algorithm searches for the parameter set of values $\left\{a_{i},\;b_{i}\right\}$ in Eqs. (1) and (2) that minimize the variance in Eq. (3). Downhill search algorithms work by following the gradient in hyperspace to a lower fit function value, using the variance defined above. The dimensions of the domain are the total number of parameters in the kernel, two times the number of exponentials, represented by the parameter set $\left\{a_{i},\;b_{i}\right\}$. For each point in this space the resulting variance serves as the fit function value. In addition, a constraint was enforced that allowed only positive values of $a_{i}$ and $b_{i}$ to be considered.

For efficiency, each image need only be converted once to RMU and then transformed to frequency space before the fitting process starts. During the iteration, the variance is repeatedly computed for parameter sets $\left\{a_{i},\;b_{i}\right\}$ generated during the downhill search. For each parameter set, the corresponding EPID image for each field size transformed to frequency space is deconvolved with the value of the frequency kernel (Eq. (2)) by simple division in the frequency domain. The result is then transformed back to the spatial domain and multiplied by the in‐air OAR. Each pixel of the resulting incident fluence image (RMU‐calibrated, deconvolved, OAR‐corrected) provides a weighting factor for the corresponding pencil beam used by the Dosimetry Check program.^(1)^ The dose to all scan points is computed and compared to the measured value for each point. The set of kernel values $\left\{a_{i},\;b_{i}\right\}$ that give the minimum variance is thus found via the downhill search algorithm. We investigated varying the number of exponentials from one to five.

### E. Reconstructing the dose for a 60° wedge

A good test of dose reconstruction using a “measured” fluence is to reconstruct the dose profile for a 60° wedge. We used the Elekta Precise 60° auto‐wedge. A $20\times 20\;{\text{cm}}^{2}$ field size image at 6 MV was acquired with the wedge in place, and the above process was used to compute the dose (RMU‐calibration, deconvolution, and multiplication by the in‐air OAR).

The wedge factor was measured with an ion chamber in water at 100 cm SSD, and then in air with a buildup cap at both 100 cm and 155 cm (155 cm is the approximate target‐to‐EPID distance). The wedge factor was also measured with the EPID by integrating the open and wedged fields for field sizes of $5\times 5\;{\text{cm}}^{2},\;10\times 10\;{\text{cm}}^{2}$, and $20\times 20\;{\text{cm}}^{2}$. The wedge factor was computed by dividing the wedged field central axis integrated pixel value by the corresponding value for the open field. The ratio was also computed after both images were deconvolved, as described above. To study the effect of beam hardening by the wedge, the wedge factor for a $10\times 10\;{\text{cm}}^{2}$ field was measured by the EPID for varying thickness of steel filtration. The target‐to‐filter distance was 85 cm.

### F. Two clinical cases

Upon image acquisition, the iViewGT software writes a separate file for each IMRT beam segment, and the scaling factor for each file is retrieved from the display. Although tedious, two clinical cases were assembled to demonstrate the feasibility of dose reconstruction for IMRT. The first case was a 10‐MV, five‐field prostate plan with a total of 42 segments. The second case was a 6‐MV, nine‐field head and neck plan with a total of 93 segments. In both cases, the treatment plan was computed by a Pinnacle treatment‐planning system (Philips Medical Systems, Andover, MA), version 6.2b, using the adaptive convolution algorithm and heterogeneity corrections.

## III. RESULTS

### A. and B. Integration of EPID images

**Figure 4:**
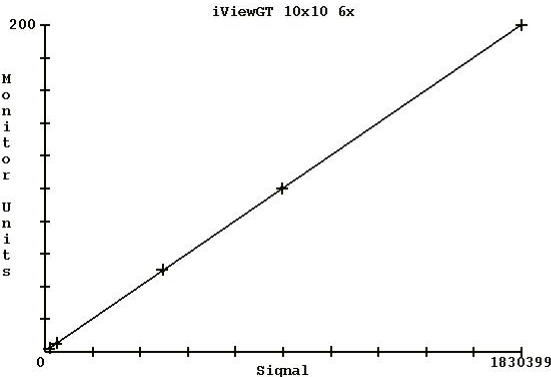
Plot of central axis integrated pixel values versus monitor units for a $10\times 10\;{\text{cm}}^{2}$ field (2 MU, 5 MU, 50 MU, 100 MU, and 200 MU with integrated pixel value of 1 830 399 for 200 MU).

A plot of the central axis integrated pixel values versus monitor units for a $10\times 10\;{\text{cm}}^{2}$ field is shown in Fig. 4. The correlation coefficient for the fit was 0.999 995. The intercept for 0 signal value was at an RMU value of 0.3. The RMU for each field was found to a standard deviation of 0.5 RMU. Application of the RMU calibration to a $10\times 10\;{\text{cm}}^{2}$ field acquired six weeks later gave the correct RMU to within 1%.

### C. and D. Conversion of EPID images to incident fluence and fitting of kernel parameters

Table 2 shows the kernel parameter fit percent error (at one standard deviation) versus the number of exponentials used in Eqs. (1) and (2). The time to fit each kernel ranged from 40 min for one exponential to 4.5 h for five exponentials on a 1.6‐GHz computer running under Windows 2000. However, these times were after a good starting solution was found from prior optimization runs. Earlier runs took as long as 9 h. Fitting the 10‐MV photon kernel with five exponentials using 6‐MV values as a starting value took 7.5 h. A good starting solution was found by first fitting two exponentials and then using that result as a starting solution with another exponential added. Once a solution for five exponentials was found, the optimization runs were repeated to produce Table 2. Some attention was needed to restrict the domain of each variable. As expected, relative improvement decreased with increasing number of exponentials, so no attempt was made to use more than five exponentials. Table 3 shows the kernel parameter values for 6 MV and five exponentials. Similar results were obtained for 10 MV.

**Table 2:** Kernel parameter goodness of fit (% error) versus the number of exponentials used in Eqs. (1) and (2). The error reported is relative to the central‐axis maximum dose at one standard deviation.

Number of exponentials	Error (%)
1	2.663
2	2.663
3	1.949
4	1.854
5	1.853

**Table 3:** Coefficients $\left\{a_{i},\;b_{i}\right\}$ for the 6‐MV point spread kernel fitted with five exponentials

Exponential	$a_{i}$	$b_{i}$
1	69.970 7	22.880 6
2	0.085 281 1	2.278 58
3	0.003 675 46	0.614 349
4	3.101 0175e‐5	0.063 886 9
5	4.561 98e‐8	0.005 741 82

Table 4 shows the percent error at 1.7 cm depth on the central axis for each field size for 6 MV. Except for the $2\times 2\;{\text{cm}}^{2}$ field size, all errors are about 1%. The measured dose for the $2\times 2\;{\text{cm}}^{2}$ field size is subject to a larger uncertainty due to the small size of the field relative to the size of the measuring ion chamber.

**Table 4:** Disagreement (% error in central axis dose at $d_{\max}=1.7\;\text{cm}$) between the measured and EPID‐calculated water phantom dose for 6 MV

Field size $\left({\text{cm}}^{2}\right)$	Error (.%)
2×2	2.6%
5×5	0.9%
10×10	0.3%
20×20	1.0%
25×25	1.1%

**Figure 5:**
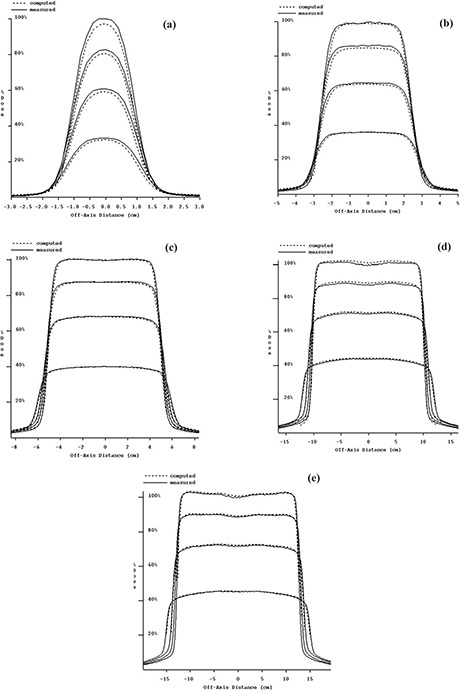
(a) to (e). Measured dose compared to EPID‐reconstructed dose and renormalized to 100% of the measured dose at $d_{\max}$. Dose profiles are at depths of 1.7 cm, 5 cm, 10 cm, and 20 cm, for 6‐MV photons. (a) $2\times 2\;{\text{cm}}^{2}$ field size. (b) $5\times 5\;{\text{cm}}^{2}$ field size. (c) $10\times 10\;{\text{cm}}^{2}$ field size. (d) $20\times 20\;{\text{cm}}^{2}$ field size. (e) $25\times 25\;{\text{cm}}^{2}$ field size.

In Fig. 5 is a plot of the measured and computed dose for each depth that was scanned. These data are normalized to 100% of the measured dose at $d_{\max}$ on the central axis for the purpose of plotting here. The computed values are also scaled such that the differences between the measured and computed curves represent differences in absolute dose.

### E. Reconstructing the dose for a 60° wedge

**Figure 6:**
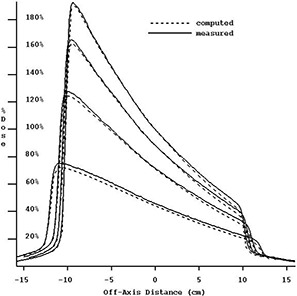
Six megavolt photon measured and EPID‐reconstructed dose profiles for a 60° wedge at depths of 1.7 cm, 5 cm, 10 cm, and 20 cm. The reconstructed absolute dose has been adjusted to agree with the measured dose. The slope of the reconstructed wedge dose profile is demonstrated. All data are normalized to 100% of the measured dose at $d_{\max}$ on the central axis.

The 6‐MV 60° wedge profile could be reconstructed. However, the EPID‐calculated central axis dose for the wedge was consistently smaller than that measured by about 16%. In Fig. 6 is the dose reconstructed for a $20\times 20\;{\text{cm}}^{2}$ field size but after forcing the computed dose to agree with the measured dose on the central axis at $d_{\max}$. Table 5 shows the wedge factors measured with the EPID and an ion chamber for both in‐water and in‐air (in‐air at 100 cm and 155 cm SCD). For 6 MV, the EPID wedge factors are consistently smaller than ion chamber wedge factors by about 16%. EPID image data processing (using integrated pixel data or reconstructed dose data) did not affect the wedge factor.

Table 6 shows the 6‐MV wedge factors measured with the EPID as a function of added steel filtration. The correct wedge factor was obtained at a steel filter thickness of 3.6 cm. The slope of the computed wedge profile was unaffected by the steel filtration.

**Table 5:** Elekta Precise 60° wedge factors (WF) for 6‐MV and 10‐MV photons. Air and water wedge factors were measured with a 0.6 cm^3^ Farmer chamber. The difference (%) between ${\text{WF}}_{\text{air},\;155\;\text{cm}}$ and ${\text{WF}}_{\text{EPID}}$ is reported in the last column.

Field size	Energy	In water	In air	In air	iViewGT	% Diff
$\left({\text{cm}}^{2}\right)$	(MV)	100 cm	100 cm	155 cm	EPID	
		$\text{SSD}+d_{\max}$	SCD	SCD		
5×5	6	0.286	0.288	0.287	0.248	15.7%
10×10	6	0.293	0.295	0.293	0.251	16.7%
20×20	6	0.306	0.307	0.304	0.260	16.9%
5×5	10	0.308	0.303	0.301	0.280	7.5%
10×10	10	0.311	0.310	0.308	0.282	9.2%
20×20	10	0.328	0.323	0.319	0.293	8.9%

**Table 6:** EPID‐derived wedge factors as a function of added steel filtration for the $10\times 10\;{\text{cm}}^{2}$ field size at 6 MV

Steel (cm)	Wedge factor
0.0	0.248
0.6	0.260
1.2	0.270
1.8	0.277
2.4	0.284
3.0	0.287
3.6	0.294
4.2	0.297

### F. Two clinical cases

**Figure 7:**
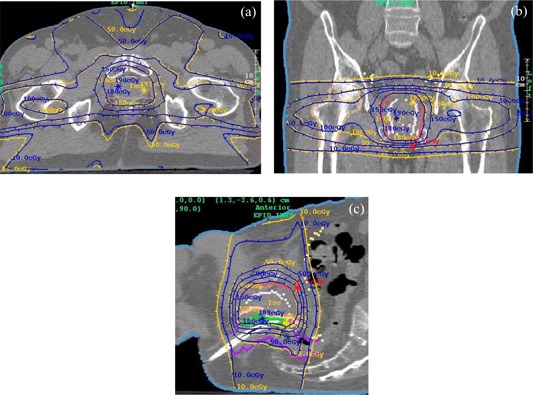
(a) to (c). Planning system dose distribution (blue) versus EPID‐reconstructed dose (yellow) for an IMRT prostate plan using 10‐MV photons. (a) Transverse plane. (b) Coronal plane. (c) Sagittal Plane.

**Figure 8:**
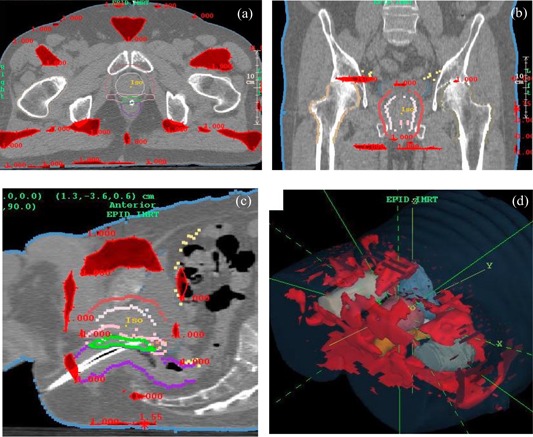
(a) to (d). Prostate plan gamma analysis for y=1.1 (tinted red), 2% dose difference (percent of maximum dose reported by Pinnacle), and 3 mm distance‐to‐agreement. (a) Transverse plane. (b) Coronal plane. (c) Sagittal plane. (d) 3D view.

Figures 7(a) to (c) show dose comparisons between the IMRT treatment plan (blue) and the EPID‐based reconstructed dose (yellow) for a 10‐MV prostate plan. The dose at isocenter agreed to within 0.1%. Figures 8(a) to (c) show the gamma distribution^(28)^ for a value of 1.0 and a corresponding dose difference of 2% or a distance of 3 mm. The gamma value is computed at each point in the planes shown by considering the radius of a sphere at which the same dose might be found, and thus constitutes a 3D search. Figure 8(d) shows a 3D, central point perspective view of the gamma isosurface of 1.0.

**Figure 9:**
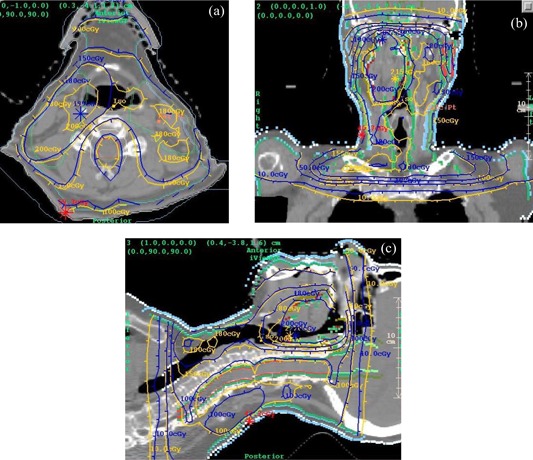
(a) to (c). Planning system dose distribution (blue) versus EPID‐reconstructed dose (yellow) for an IMRT head and neck plan using 6‐MV photons. (a) Transverse plane. (b) Coronal plane. (c) Sagittal plane

**Figure 10:**
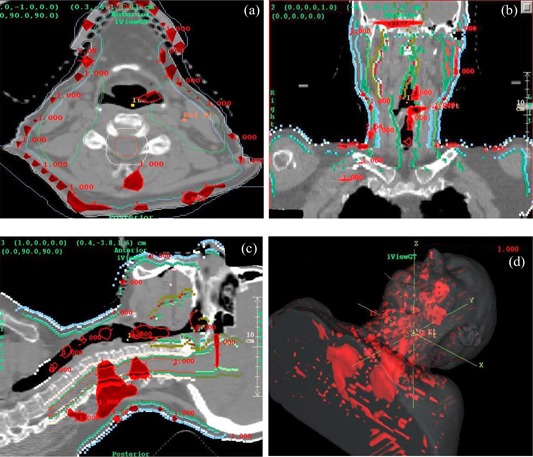
(a) to (d). Head and neck gamma analysis for γ=1.0 (tinted red), 4% dose difference (percent of maximum dose reported by Pinnacle), and 2 mm distance‐to‐agreement. (a) Transverse plane. (b) Coronal plane. (c) Sagittal plane. (d) 3D view.

In Figures 9 (a) to (c) are shown the dose comparisons for the 6‐MV, IMRT head and neck case. The dose at isocenter agreed to within 2.1%. The gamma distribution for this plan is shown in Figs. 10 (a) to (d). For a gamma value of 1.0 the dose difference was 4%, and the distance‐to‐agreement was 2 mm.

## IV. DISCUSSION

While the iViewGT EPID can be used to reconstruct 3D dose accurately, clinical use for IMRT QA is still laborious. The pixel scaling factors are not included in the DICOM export from the iViewGT station. Thus, the user must manually record the individual segment pixel factors and then combine the segments using the Dosimetry Check software. As a result, the time required to perform 3D dose reconstruction with the EPID is probably the same as the established method using film. Yet, EPID QA has advantages over film QA in that it is linear, more reproducible, and eliminates film processing problems.

Nevertheless, we have demonstrated acceptable results on two IMRT plans. In homogeneous regions the 3D reconstructed isodose curves are essentially the same as the planned isodose curves. Discrepancies between planned and reconstructed dose, particularly in the head and neck case, are to be expected for at least two reasons. First, Dosimetry Check uses a pencil beam algorithm, whereas Pinnacle uses the adaptive convolution/superposition algorithm. Convolution/superposition is generally known to be more accurate at tissue interfaces where electronic equilibrium is perturbed.^(29)^ Given that Dosimetry Check is a QA tool, these differences are not considered critical. Second, Pinnacle version 6.2b does not explicitly model either multileaf collimator leaf‐end leakage or inter‐leaf leakage. Increasing the number of segments in a plan, as in a head and neck case, will readily demonstrate any inaccuracy of the planning system. Leaf‐end leakage and inter‐leaf leakage are modeled in Pinnacle version 7.4.

The open‐field deconvolution kernel is unable to accurately reproduce the wedge factor and, thus, the dose from a wedged field. It was demonstrated here that the correct wedge factor can be obtained if both the open and wedged beams are heavily filtered, which affirms that this problem has something to do with alteration of the energy spectrum. It appears that the iViewGT EPID may be underresponding to the wedge‐hardened beam. Indeed, supporting these findings, Parent et al.^(30)^ also found that beam‐hardening results in an underresponse by an a‐Si EPID.

Despite the spectral changes occurring along the heel‐toe direction, the deconvolution kernel does reproduce the correct slope of the wedge profile (Fig. 6). This agreement may be explained by the following. Over the length of the $20\times 20\;{\text{cm}}^{2}$ field, in the heel‐toe direction, there is a common, uniformly thick amount of the wedge intercepted by the beam. Above this common thickness is where the “wedging” occurs. Smaller fields have a thicker amount of common filtration than larger fields. This common thickness is thought to be sufficiently hardening the beam, even at the toe end, such that the EPID correctly reproduces the slope of the wedge profile.

If this verification system were to be used with fields that include a wedge, or any partial attenuator such as a half‐height block, then the system would have to be used with a large amount of filtration and a deconvolution kernel generated specifically for a filtered beam. This could present some mechanical difficulties in putting such a filter in the beam for all measurements, but the filter could be mounted in the blocking tray slot. Chang et al.^(11)^ did not find a spectral difference between ion chamber measurements and those using the Varian Mark I/II EPID for beams attenuated by lead up to 6 cm thick for 15 MV. Therefore, this problem may not be common to all EPIDS.

## V. CONCLUSION

In this study we examined some of the problems in using an EPID to generate in‐air incident fluence distributions for 3D dose reconstruction.

The conversion of raw integrated pixel values to RMU was linear and reproducible for the EPID employed here. Because the intercept is nearly zero, it is sufficient to use only one exposure to generate the RMU calibration curve. The application of the EPID should be superior to film in both ease of use and reproducibility.

Fitting a mathematical form for the kernel was demonstrated to be a feasible process. The dose from open fields was successfully reconstructed using the resulting kernel. The mathematical form used for the kernel was derived for a different EPID but was sufficient for the iViewGT EPID. The mathematical form therefore shows promise of being applicable to other EPIDs.

Comparison to measured dose in water was successfully employed here as the means to fit the parameters for the kernel. Using the sum of exponentials as the mathematical form for the kernel was successful in converting the iViewGT EPID images to fluence for the reconstruction of the dose for open fields. Further, because the kernel was fitted, we avoided both detailed analysis of EPID construction and Monte Carlo simulations necessary to determine a kernel. Fitting the kernel in this manner minimizes the error in the intended use of the EPID for dose reconstruction and so improves the utility of the method.

It was sufficient to multiply the deconvolved image with the measured in‐air off‐axis ratio to restore the “horns” that were removed by the EPID calibration procedure.

The slope of a wedged field was successfully reconstructed. The problem of obtaining the correct absolute dose under the wedge is not a problem with the kernel and reconstruction process but rather a problem with the EPID energy response. The construction of a particular EPID and its energy response characteristics may therefore influence whether it can be employed for dosimetry as described in this report. Care must be taken when investigating the use of an EPID for dosimetry with the use of attenuators such as wedges because of the possible problem with the energy response of the EPID. Inserting a large amount of filtration to enable the use of an EPID for all clinical situations is a possible solution. It was beyond the scope of this study to consider the EPIDs currently available and to consider their suitability separately for this application.

The two clinical cases presented here demonstrate that the fitted kernel can reconstruct an IMRT case to sufficient accuracy for dose delivery verification. Upon improving the image file transfer process to include pixel scaling information, it will be feasible to perform patient 3D dose reconstruction on a routine basis.
